# Treating Autoimmune-Related Interstitial Lung Disease With B Cell Depletion

**DOI:** 10.3389/fmed.2022.937561

**Published:** 2022-06-30

**Authors:** Stamatis-Nick C. Liossis, Constantina A. Bounia

**Affiliations:** ^1^Division of Rheumatology, Patras University Hospital, Patras, Greece; ^2^Department of Internal Medicine, University of Patras Medical School, Patras, Greece

**Keywords:** interstitial lung disease, connective tissue diseases, B cell depletion, systemic sclerosis, rheumatoid arthritis

## Abstract

Autoimmune rheumatic diseases may affect vital organs with lung involvement being severe and difficult to treat manifestation. Systemic sclerosis (SSc) commonly affects the lung in the form of interstitial lung disease (ILD). ILD may be also seen in patients with rheumatoid arthritis (RA), Sjögren's syndrome (SS), systemic lupus erythematosus (SLE), inflammatory myositis (IM), antisynthetase syndrome (AS), and the ANCA-associated vasculitides (AAV). Rituximab (RTX) is an anti-CD20 B lymphocyte depleting mAb, often administered in the treatment of autoimmune rheumatic diseases. Although RTX is an off-label treatment for CTD–ILD, there are numerous reports providing data that is effective in improving both pulmonary function tests (PFTs) and chest computed tomography findings consistent with ILD. There are retrospective uncontrolled studies that assess RTX as a treatment of ILD in autoimmune diseases. These studies, apart from one, do not include patients with AAV-ILD. In SSc-ILD, in particular, there are both controlled and uncontrolled studies displaying encouraging results following B cell depletion. In addition, a number of retrospective uncontrolled studies and fewer prospective studies evaluate RTX in connective tissue diseases CTD–ILD. Although RTX is an approved treatment for AAV there are scarce only data focusing on patients with AAV-ILD specifically. The results of a handful of studies comparing treatment of CTD-ILD with RTX to treatment with other agents are in favor of RTX. Results from large, still ongoing controlled trials are awaited to ascertain RTX effects in ILD encountered in autoimmune rheumatic diseases. We review herein the results of the different RTX trials in patients with autoimmune disease–associated with ILD. Despite the heterogeneity of these studies, RTX may be considered an alternative and safe but still off-label treatment for patients with refractory CTD–ILD.

## Introduction

**Figure 1:**
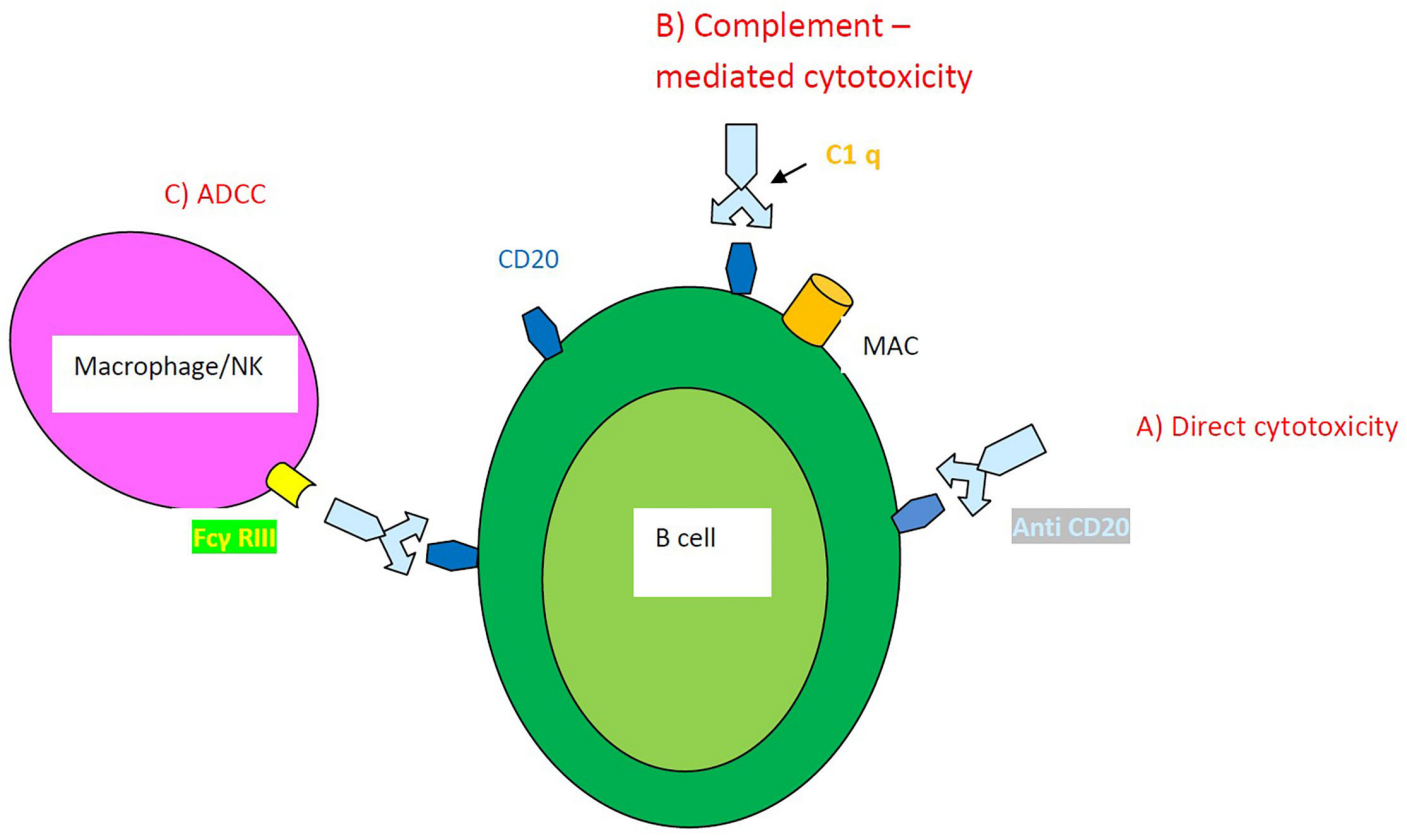
Mechanisms of action of RTX-induced B cell depletion. **(A)** Direct cytotoxicity **(B)** Complement–mediated cytotoxicity **(C)** Antibody-dependent cell-mediated cytotoxicity ADCC.

Interstitial lung diseases (ILD) are a group of parenchymal lung disorders sharing clinical and radiological phenotypes. CTDs such as systemic sclerosis (SSc), rheumatoid arthritis (RA), systemic lupus erythematosus (SLE), Sjögren's syndrome (SS), inflammatory myositis [polymyositis (PM), dermatomyositis(DM), and antisynthetase syndrome (AS)] may affect lung parenchyma (1). In addition, ANCA—associated vasculitides (AAV) may present with ILD. CTD-ILD is divided

into seven histological types including usual interstitial pneumonia (UIP), nonspecific interstitial pneumonia (NSIP), desquamative interstitial pneumonia (DIP), respiratory bronchiolitis (RB), organizing pneumonia (OP), diffuse alveolar damage (DAD), and lymphoid interstitial pneumonia (LIP). NSIP and UIP are the most common ILD subtypes encountered in patients with connective tissue diseases (CTDs) (2). Although treating lung involvement in CTDs has been extensively studied it is still a challenge to improve the prognosis of these patients (3, 4). Immunosuppressive agents currently represent the standard of care for patients with CTD-ILD. SSc beholds the majority of studies assessing the effects of immunosuppressants and also of studies regarding RTX administration in ILD (5). RTX is a monoclonal antibody against the surface marker CD 20 of B lymphocytes. RTX depletion of B lymphocytes is achieved in three ways (Figure 1): (a) anti-CD20 antibody induces internal signaling within the B cell, causing antiproliferative effects or cell death (direct cytotoxicity) (b) the first component of complement (C1) binds to the Fc portion of the anti-CD20 resulting in the activation of the complement cascade and cell lysis through the formation of membrane attack complexes (MAC) of the B cell (complement-dependent cytotoxicity) and (c) the effector cells such as natural killer cells or macrophages bind to the Fc portion of the anti-CD20 molecule through their Fc γ RIIIa receptors (antibody-dependent cell-mediated cytotoxicity-ADCC) (6).

**Figure 2:**
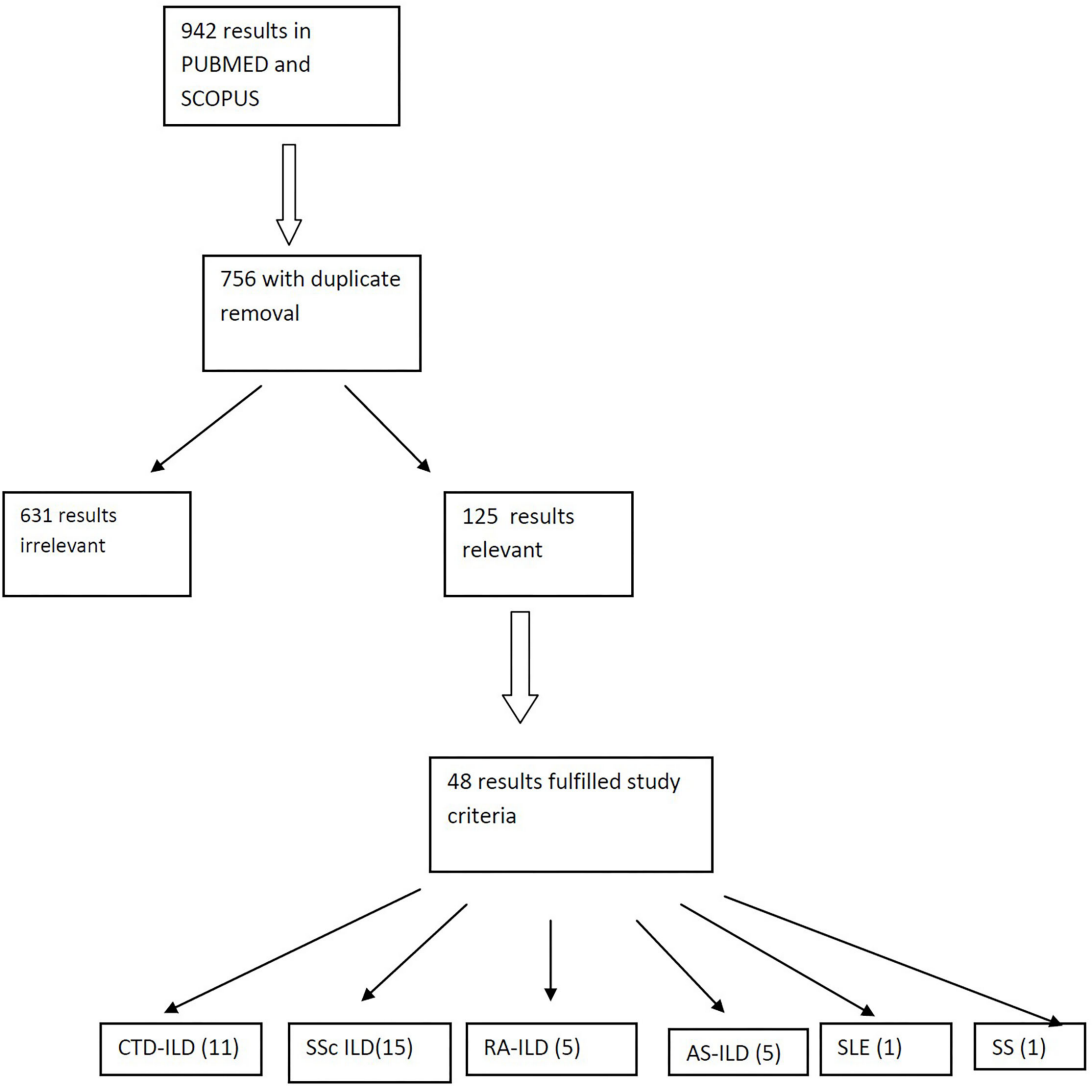
Flow chart and strategy of the studies researched and assessed.

We searched the PUBMED and SCOPUS using the following terms: interstitial lung disease, rheumatic diseases, connective tissue diseases, systemic sclerosis, rheumatoid arthritis, antisynthetase syndrome, Sjogren's syndrome, systemic lupus erythematosus, Anca-associated vasculitis, rituximab, and B-cell depletion. Preference was given in clinical trials, randomized controlled trials and metanalyses over the past 10 years. Reviews and case reports were not included (Figure 2). In this review, we discuss the results of studies evaluating RTX as an alternative treatment for ILD in patients with CTDs either collectively in groups of patients with different CTDs or as a separate group with a single CTD. All studies evaluate the changes of pulmonary function tests (PFTs) like forced vital capacity (FVC) and diffusing lung capacity for carbon dioxide (DLCO) and fewer studies examine for alterations of lung parenchyma in chest high resolution computed tomography (HRCT) following treatment of patients with RTX.

## Retrospective Studies in CTD–ILD

Duarte et al. evaluated 48 patients with CTD–ILD (7); 30 patients with RA (two of them with secondary SS), four with pSS, four with SLE, three with SSc, two with overlap SSc/PM, two with PM, twowith AS, one with DM, and one with an overlap of SLE/pSS. Most patients initiated RTX treatment because of lung involvement and a few were already on RTX treatment for other disease manifestations. Thirteen patients had already received other immunosuppressants for ILD such as cyclophosphamide (CYC) (*n* = 10) followed by mycophenolate mofetil (MMF) (*n* = 5 out of 10) or azathioprine (AZA) (*n* = 5 out of 10), and AZA as first choice (*n* = 3). RTX as the initial treatment for ILD was administrated in 35 patients. All patients were evaluated with PFTs and HRCTs performed at 6 and 12 months following RTX administration for a 3-year period of follow-up. At 12 months, DLCO values remained stable (mean: + 5.4%, *p* = 0.12) compared to baseline values and FVC improved (mean + 4.3%, *p* = 0.03) compared to baseline values. When the patients were separately evaluated according to NSIP or UIP pattern in HRCT, the following were observed: in patients with NSIP DLCO values slightly, but not significantly, increased at 12 months after RTX (8.5%, *p* = 0.08), while FVC values increased significantly (4.5%, *p* = 0.04) compared to baseline. However, patients with UIP had no significant change at 12 months after RTX compared to baseline either in DLCO (2.5%, *p* = 0.77) or in FVC (4.2%, *p* = 0.16). Potential changes in HRCT ILD pattern and extent were not addressed.

Lepri et al. retrospectively enrolled 44 patients in their study (23 with SSc, 15 with AS, and 6 with MCTD) (8). All patients were diagnosed with ILD and were treated with RTX. Most patients (15/23 with SSC, 8/15 with AS, and 5/6 patients with MCTD) were also treated with other DMARDs. At 12 months after RTX administration patients with SSc displayed a statistically non-significant increase of FVC from 81.0% at baseline to 89.0%. Patients with AS displayed a stabilization of FVC (53% at baseline vs. 51.4% at 12 months) similar to the group of patients with MCTD (64.5% at baseline vs. 63% at 12 months). DLCO values in the SSc group increased minimally (61% prior RTX to 63% at 12 months) and were considered as stable at 2 years of treatment compared to baseline levels. Similar results were reported for patients with AS a non-significant increase of DLCO from 41.7% at baseline to 52% at 12 months of treatment with RTX and stability of DLCO at 2 years of treatment with RTX compared to baseline levels before RTX administration. Finally, patients with MCTD had no significant changes at 1 year but displayed a trend in the improvement of DLCO at 2 years following treatment with RTX compared to baseline.

Atienza-Mateo et al. enrolled in their retrospective study 26 patients with autoimmune diseases that were treated with RTX due to lung involvement (9). Patients were diagnosed with SSc (*n* = 7), IIM (*n* = 6), ASS (*n* = 6), amyopathic DM (*n* = 1), RA (*n* = 5), interstitial pneumonia with autoimmune features (IPAF) (*n* = 3), pSS (*n* = 3), and myeloperoxidase anti-neutrophil cytoplasmic antibody (MPO-ANCA) positive (*n* = 2). Nineteen out of 26 patients were on immunosuppressive therapy [MMF, hydroxychloroquine (HCQ), methotrexate (MTX), AZA] and 3/26 patients were on antifibrotic therapy at the time of enrollment. RTX was administrated 1,000 mg biweekly (*n* = 20) (“RA scheme”), 500 mg biweekly (*n* = 4), and 375 mg/m^2^ weekly for 4 weeks (*n* = 2) (“lymphoma scheme”). The study reported a slight improvement of all PFTs (FVC, FEV1, and DLCO) that was preserved after 6, 12, and 24 months of treatment with RTX compared to baseline levels. In addition, a significant increase in DLCO values was observed at 12 months compared to baseline (mean ± SD: 34.02 ± 14.75% at baseline vs. 38.22 ± 15.86% at 12 months, respectively, *p* = 0.025). Lung involvement was also evaluated with chest HRCT in 23 out of 26 patients. Interstitial lung involvement was unchanged in 15/23 patients (65.2%), worsened in 5/23 patients (21.7%) and improved in 3/23 patients (13.1%). This study finally emphasizes that RTX unlike other previous studies was administered early in ILD diagnosis before any worsening of ILD disease was confirmed.

Sharp et al. studied retrospectively 24 patients with rheumatic diseases that developed ILD refractory to conventional therapy and therefore received RTX (10). The trial included 10 patients with AS, 3 with DM, 3 with SSc, 2 with SS, 2 with SLE and 4 with unclassifiable CTD-ILD. RTX was administered in the “RA scheme” and PFTs and HRCT images were evaluated before and after 6 months of treatment with RTX. FVC improved with a mean change of 4.1%, (*p* = 0.01) at 6 months after RTX compared to baseline, and DLCO did not change significantly. HRCT imaging after treatment with RTX revealed a non-significant mean change of disease extent of −3.75% compared to baseline; it should be noted that HRCT depicted deterioration in 9/22 patients following treatment with RTX.

Robles-Perez et al. evaluated 18 patients with rapidly progressive ILD awaiting in a list for lung transplantation (11); progressive ILD was defined as an FVC < 60% or worsening FVC > 10% in the last 6 months and/or DLCO < 40%. The patients included were 7 with SSc, 5 with RA, 4 with SLE, 1 with SS and 1 with AS. All patients received RTX 1,000 mg biweekly as an add-on therapy on top of immunosuppressive therapy and were evaluated with PFTs and HRCTs at time-points 0, 12 and 24 months after treatment with RTX. FVC increased significantly (+6.3%) at 12 months after RTX compared to baseline (*p* = 0.033) and DLCO values also clearly increased (+12.4%) at 12 months after RTX compared to baseline values *p* ≤ 0.001. Ten out of 18 patients were evaluated 2 years following treatment with RTX and they again displayed a significant increase of DLCO compared to baseline levels (+ 15.3%, *p* = 0.001). In contrast, FVC values did not change significantly 2 years after treatment with RTX compared with pre-treatment levels in these 10 patients. Thirteen patients also underwent chest HRCT before and after 1 year of RTX administration. Stabilization or improvement was seen in 10/13 patients (76.9%) when scored by two independent radiologists and deterioration was reported in 3/13 patients.

Keir et al. analyzed 50 patients with ILD 33 of which had CTDs (12). Patients with CTDs included 10 with IIM, 8 with SSc, 9 with UCTD, 2 with MCTD, 2 with RA, 1 with SLE, and 1 with SS. All patients had advanced ILD with a mean FVC of 44.0% and a mean DLCO of 24.5%. In addition, they all had resting state hypoxia (mean PaO2: 8.3 kPa). RTX was administered in the “RA scheme” resulting in a median improvement of FVC of 6.7% (*p* < 0.01) and a stabilization of DLCO with a median change of 0% (*p* < 0.01) at 6 and 12 months of RTX treatment compared to baseline levels. Patients (*n* = 50) were considered as responders according to stabilization (*n* = 22) or improvement (n=14) of PFTs and as non-responders (*n* = 14) due to deterioration of PFTs. Comparison between the two categories depicted a lesser decline of FVC at 6–12 months after RTX compared to baseline in responders (−12.9%) vs. non-responders (−20.0%) after 6 to 12 months of RTX compared to baseline, (*p* = 0.05).

However, Zhu et al. conducted a retrospective controlled trial comparing the effects of combined RTX plus MMF treatment in patients with CTD-ILD (*n* = 15) (RTX group) vs. MMF treatment alone (*n* = 68) in patients with CTD-ILD (control group) (13). The evaluation of PFTs between the two groups reveals the following: the absolute change in FVC (% predicted, post-treatment—baseline) was −3.0 in the RTX group thus showing a significant decrease. On the other hand, the control group had a significant increase in the absolute change of FVC of + 2.0, *p* = 0.03. Moreover, the absolute change of DLCO was significantly decreased at −3.0 in the RTX group, whereas it improved significantly at + 4.5 in the control group, *p* = 0.046. The mixed model analysis performed however in the two groups did not display a significant difference in both PFTs over time. HRCT scores showed no significant differences between the RTX group (17.5 ± 4.6) compared to the control group (12.6 ± 4.4). It should be noted that the steroid dosages decreased in the RTX group, whereas it did not in the control group (*p* = 0.017). The mortality rate after treatment was calculated at 3/15 (20.0%) in the RTX group vs. 7/68 (10.3%) in the control group. Results of this trial do not favor the addition of RTX on top of standard of care treatment. The authors point-out that patients in the RTX group had longer disease duration and lower DLCO percentages at baseline.

## A Prospective Trial of B Cell Depletion in Patients With CTD–ILD

Mena-Vazquez et al. evaluated 37 patients with CTD-ILD that received RTX as add-on therapy upon the deterioration of either clinical symptoms or PFTs compared to the time of diagnosis (14). Nineteen patients with RA (51.4%), 14 with SS (37.8%), and 4 with IM (10.8%) received RTX in the “RA scheme” and were evaluated before and 12 months after treatment. The above parameters improved (*n* = 6) or stabilized (*n* = 7) in 62.2% of patients and worsened (*n* = 7) or died (*n* = 7) in 37.8% of patients.

In the whole group of patients on RTX or in any disease-specific subgroup mean PFT and DLCO values did not decrease significantly during the first 12 months of treatment with RTX compared with baseline. HRCT revealed radiological progression in 14/37 patients (37.8%) while in 16/37 patients (43.2%) revealed stabilization and in 7/37 (18.9%) revealed improvement, but differences were not significant.

Table 1 summarizes the studies of RTX use in CTD-ILD.

**Table 1:** Trials of RTX use in CTD-ILD.

**References**	**No of patients**	**Disease**	**RTX scheme**	**Follow up**	**Outcomes**	**Safety of RTX**
Duarte et al. (7)	30	CTDs	RA scheme	3 y	Improved FVC Stable DLCO	13/30 pts discontinued RTX 4/13 due to infection
Lepri et al. (8)	44	CTDs	RA scheme	1y/2y	Stable FVC and DLCO	12/44 pts serious AE
Atienza-Mateo et al. (9)	26	CTDs	RA scheme (*n* = 20) Lymphoma scheme (*n* = 2)	2 y	Stable PFTs at 6 m, 1, 2 y ↑DLCO at 1 y stable HRCT	6/26 discontinued RTX due to AE 3/26 death
Sharp et al. (10)	24	CTDs	RA scheme	6 m	Improved FVC stable DLCO 13/22 stable HRCT	No serious AE
Robles-Peres et al. (11)	18	CTDs	RA scheme	1y/2y	Increased PFTs at 1 y 10/18 stable HRCT at 1 y	No serious AE
Zhu et al. (13)	15 RTX vs. 68 non-RTX	CTDs			Significant DLCO change and steroid reduction vs. control Similar FVC, HRCT score vs. control	No serious AE
Keir et al. (12)	33	CTDs	RA scheme	6 m/1 y	Improved FVC, stable DLCO	–
Mena-Vazquez et al. (14)	37	CTDs	RA scheme	1 y	Stable PFTs HRCT stable or improved	29/37 lung infection, 7/27 deaths

*No, Number; m, month; y, year; AE, adverse events; CTDs, connective tissue diseases; RTX, rituximab; PFTs, pulmonary function tests; FVC, forced vital capacity; DLCO, diffusing lung capacity for carbon monoxide; HRCT, high resolution computed tomography; RA, rheumatoid arthritis. ↑, Increased*.

## Ongoing Clinical Trials of RTX in Patients With CTD-ILD

An ongoing, multicentre, prospective, randomized, double-blind, controlled trial (RECITAL) compares RTX vs. CYC in the treatment of patients with ILD in CTD such as SSc, IIM (including AAS), or MCTD (15). RTX will be administered in the “RA scheme” and CYC will be administered IV at 600 mg/m^2^ body surface area monthly. A total of 116 patients is expected to be randomized 1:1 to each of the two treatment arms in this 48-wk trial. The primary end-point is the change in FVC at 24 weeks and secondary endpoints include treatment safety, changes in FVC at 48 weeks as well as survival, change in oxygen requirements, and total 48-wk corticosteroid exposure.

Another multicentre, prospective, randomized, double-blind, placebo controlled, superiority trial evaluates the efficacy and safety of RTX plus MMF in patients with ILDs (EvER-ILD) (16). A broad range of patients with non-responding, resistant to previous therapy ILD was recruited to receive different treatment schemes; one group (*n* = 61) will receive RTX (“RA scheme”) plus MMF, while the other group (*n* = 61) will receive one placebo infusion plus MMF for 6 months. Pulmonary function, with the changes of FVC as the primary endpoint, will be evaluated at 6 months.

## A Meta-Analysis of Patients With CTD-ILD

In their meta-analysis, Xing et al. selected 6 retrospective studies in which 242 patients with CTD-ILD had been included (17). Five out of 6 studies included patients with SSc-ILD and 1 included patients with RA-ILD. All patients were analyzed for potential changes in the PFTs after being treated with RTX. The FVC was evaluated in 210 patients from all 6 studies. Analysis disclosed that the mean difference of FVC between RTX and conventional therapy was 5.4 (95% CI 3.73 to 7.08; *p* < 0.00001) in favor of RTX. However, DLCO was evaluated in 111 only patients from four studies (due to the high heterogeneity of results). The mean difference between post-treatment RTX and conventional therapy was-−2.22 (95% CI −6.83–2.40) in favor of conventional treatment though it was not statistically significant. This meta-analysis suffers from high heterogeneity between the studies included; it mainly refers to SSc-ILD and not ILD due to other CTDs. In addition, the very small number of studies included represents another limitation.

## Systemic Sclerosis SSC

### Prospective Controlled Studies in Patients With SSc-ILD

A controlled study by Daoussis et al. evaluated 8 patients with SSc-ILD who received RTX as an add-on treatment vs. 6 patients with severe SSc-ILD who continued their standard treatment (18). Patients in the RTX group demonstrated a significant increase of FVC compared to baseline values (mean ± S.D.: 68.13% ± 19.69 vs. 75.63% ± 19.73, at baseline vs. 1 year, respectively, *p* = 0.0018). DLCO increased significantly as well (mean ± S.D.: 52.25 ± 20.71 vs. 62 ± 23.21, at baseline vs. 1 year, respectively, *p* = 0.017). No significant changes in PFTs of the control group were observed. Chest HRCT scores, based on a semi-quantitative program, disclosed no worsening at 24 weeks in the RTX group, but patients in the control group displayed a slight worsening in HRCT involvement scores at 24 weeks.

Jordan et al. studied 25 patients with SSc-ILD initiating RTX vs. 25 patients with SSc-ILD that were not treated with RTX from the EUSTAR database (19). Patients in the RTX-group at 6 months displayed a stable FVC (60.6 ± 2.4 vs. 61.3 ± 4.1%; *p* = 0.5) and a significantly increased DLCO (41.1 ± 2.8 vs. 44.8 ± 2.7%; *p*=0.03) when compared to baseline values. DLCO did not change significantly in a direct comparison between the two groups. A comparison between the RTX group *vs*. the non-RTX group revealed significant changes in percentages of predicted FVC (0.4 ± 4.4 vs. −7.7 ± 3.6; *p* = 0.02) and in the absolute FVC values changes (0.8 ± 2.2 vs. −4.8 ± 1.7; *p* = 0.01) as well.

A multicentre controlled trial in Greece enrolled 51 patients with SSc-ILD receiving RTX vs. 33 patients with SSc-ILD receiving standard treatment, for a period of 4 years (20). FVC in the RTX group during the first 2 years significantly increased (mean ± SD of FVC: 80.6 ± 21.21 vs. 86.90 ± 20.56 compared to baseline *p* = 0.041, but the patients receiving standard treatment displayed no significant changes in FVC. In addition, patients treated with RTX for 7 years (*n* = 5) had numerically, but not significantly higher FVC compared to baseline (mean ± SD of FVC: 91.60 ± 14.81 vs. 86.90 ± 20.56, *p* = 0.158). However, the patients (*n* = 9) on standard treatment had significant FVC deterioration at the 7th year of follow-up (*p* < 0.01). Moreover, a direct *comparison between the two groups revealed a significant benefit for the RTX group vs*. the non-RTX group (*p* = 0.013). Patients in the RTX group preserved their DLCO values for the 7-yr period, but patients in the non-RTX group displayed a significant reduction of their DLCO values (*p* = 0.004).

Boonstra et al. studied 16 patients with early SSc for a 2-yr follow-up period; one group was treated with RTX (*n* = 8) with the “RA scheme” and the control group (*n* = 8) with placebo (21). Previous immunosuppressive therapy was allowed for all patients during the 2 yrs of follow-up. FVC and the extent of lung involvement slightly but non-significantly improved with RTX after 2 years; FVC (placebo: −1.4 vs. RTX: +4, *p* = 0.65), and DLCO% (placebo: −2.2, RTX: −6.0, *p* = 0.77). Analysis of HRCT lesions according to criteria set by Goh disclosed a mean change in the percentage of affected lung tissue between baseline and 12 months of −1.6% for the RTX group and +2.8% for the placebo groups (*p* = 0.28).

The EUSTAR study evaluated 146 out of 254 patients with SSc-ILD who received RTX plus standard treatment vs. standard treatment alone, for a 2-year period (22). More specifically, Elhai et al. studied 254 patients from the EUSTAR database with SSc-ILD treated with RTX from a pool of 9,575 patients with SSc-ILD receiving standard treatment for a period of 2 years. During the 2 years of evaluation in both groups FVC, as well as DLCO values, remained practically stable. RTX-treated vs. standard-treated patients did not have significantly different rates of decrease in FVC > 10% [6.5 vs. 6.6 events per 100 person-years; OR: 1.03 (0.55–1.94); *p* = 0.93] and in DLCO as well. However, the patients in the RTX group discontinued or reduced the daily dosages of steroids earlier [OR: 2.34 (1.56–3.53), *p* < 0.0001]. Even though the number of patients was large enough, the trial results may have been confounded by patient heterogeneity. There were differences in the extent of lung involvement of enrolled patients, in the chronicity of the disease, and even in the RTX administration protocol among the various participating centers to such an extent that may preclude the extraction of safe conclusions. In secondary analyses of PFTs patients treated with RTX plus MMF (*n* = 37) showed better outcomes as compared with patients receiving RTX alone [delta FVC in RTX plus MMF: 5.22 (0.83–9.62); *p* = 0.019 vs. delta FVC: 3 (0.66–5.35); *p* = 0.012 in patients receiving rituximab alone].

In a recent, open-label, prospective, randomized, controlled trial the authors compared head-to-head RTX vs. monthly cyclophosphamide (CYC) treatment (23). Sixty patients with early, diffuse SSc with ILD and anti-Scl70(+), were enrolled to receive CYC or RTX as first-line treatment. Patients in the CYC treatment arm received 500 mg/m^2^ CYC IV pulses every 4 weeks for 24 weeks. Patients in the RTX group received RTX as in the “RA scheme.” The RTX group revealed an improvement of FVC% at the end of 6 months when compared to baseline values (RTX group: 61.3–67.5%, *p* = 0.002) while the CYC group did not (CYC: 59.3–58.1%, *p* = 0.496). RTX turned out to be safer than CYC regarding cases of malignancy, gangrene, and ovarian failure, but it displayed higher rates of (minor) infusion reactions. Based on these data RTX may be considered a first-line therapy instead of CYC especially if we take into account the safety demonstrated too in this trial. However, one should take into account that the current standard-of-care treatment for SSc-ILD is MMF and not CYC. The controlled trials of RTX effect on SSc-ILD are depicted in Table 2.

**Table 2:** Controlled trials of RTX in SSc-ILD.

**References**	**No of patients**	**Disease**	**RTX scheme**	**Follow up**	**Outcomes**	**Safety of RTX**
Daoussis et al. (18)	8 RTX vs. 6 Control	SSc	Lymphoma scheme	1 y	Improved PFTs and stable HRCT score in favor of RTX	3 serious AE/2 lung infections, 1 infusion reaction
Jordan et al. (19)	25 RTX vs. 25 control	SSc	RA scheme	6 m	Increased FVC, stable DLCO in RTX group	11/53 infections No other serious AE
Daoussis et al. (20)	33 RTX vs. 18 control	SSc	Lymphoma scheme	7 y	Improved FVC, stable DLCO vs. control	3 serious infections, 2 infusion reactions, 5 deaths
Boonstra et al. (21)	8 RTX vs. 8 con	SSc	RA scheme	2 y	Stable PFTs and HRCT similar to control group	7 serious AE in RTX(infusion reaction) vs. 4 serious AE in control (weight loss)
Eustar et al. (22)	254 RTX vs. 9,575 control	SSc	RA scheme (*n* = 203) lymphoma scheme (*n* = 4) else (*n* = 27)	2 y	Stable PFTs similar to control/steroid reduction vs. control	36/254 serious AE, 24/254 discontinue, 6 deaths unrelated
Theibaut et al. (24)	23 RTX vs. 26 non-RTX	SSc	RA scheme	2 y	Improved PFTs	Infusion reactions, mild infections/2 deaths
Sircar et al. (23)	30 RTX vs. 60 CYC	SSc	RA scheme	6 m	Improved FVC on RTX vs. control	RTX AE 9/30 vs. CYC AE 21/30

### Prospective Uncontrolled Studies in Patients With SSc-ILD

Lafyatis et al. studied 15 patients with early SSc treated with RTX only (“RA scheme”) without concomitant disease modifying anti-rheumatic (DMARD) therapy (25); they did not find a clear beneficial effect on skin fibrosis and pulmonary function at 6 and 12 months of follow-up. Patients with severe lung disease were excluded from this trial; therefore this might explain why the average FVC and DLCO values did not change significantly at 6 months. In detail, baseline FVC changed from 89.2 to 92.7% after RTX treatment and DLCO changed from 79.7% at baseline to 77.9% after RTX administration. In agreement with the above, little or no progression of pulmonary disease was depicted on HRCT. Because patients included in this study had a near-normal respiratory function, no safe conclusions can be drawn regarding the effects of B cell depletion treatment.

Daoussis et al. presented a beneficial effect of RTX (“lymphoma scheme”) when the follow-up of our initial cohort of 8 patients that were previously reported at 1 year was extended to 2 years of treatment (26). FVC values displayed a significant improvement at 2 years (mean ± SEM: 77.13 ± 7.13 vs. 68.13 ± 6.96, respectively, *p* < 0.0001) as did the DLCO values (mean ± SEM: 63.13 ± 7.65 vs. 52.25 ± 7.32, respectively, *p* < 0.001). Moreover, semiquantitatively ground glass lesions were depicted in HRCT of 5/8 patients. The results are considered encouraging, even though the numbers of patients enrolled were small, RTX was administered concurrently with other immunosuppressing drugs (MMF in the majority) and there is not a control group in this study.

Another open-label study by Smith et al. in patients with early diffuse SSc who received RTX for a 2 year period, displayed a statistically significant overall decrease of FVC percentages, with a mean FVC of 92.8% at baseline vs. 84.7% at 24 months (*p* = 0.047) (27). However, no clinical worsening was DLCO values remained stable over the 2-years.

Bosello et al. administered RTX (“RA scheme”) in 20 patients with early (<3 years) and extensive SSc-ILD (28). During a follow-up period of 2 years, the evaluation of PFTs every 6 months and of chest HRCT every 12 months indicated no significant changes in FVC, DLCO, and chest HRCT lesions over time. However, there was marked heterogeneity regarding the duration of follow-up and the number of RTX cycles administered. When analyzed separately, the patients with the restrictive disease (*n* = 6) in PFTs (representing perhaps the most interesting patient subset) had an increase in their FVC but insignificant changes in their DLCO and HRCT scores during the first year of RTX treatment. In contrast, patients without restrictive disease in PFTs (*n* = 8) displayed no increases in the above parameters throughout the study.

Fraticelli et al. studied 15 patients with SSc-ILD receiving RTX for 6 months followed by MMF administration escalated to 2 gr for 6 months (29). After 6 months of RTX, there was a significant increase in FVC (*p* = 0.0197) and FEV1 (*p* = 0.0278) compared to baseline. The HRCT pattern was analyzed with two different methods. The first was a semiquantitative standardized method, but the results did not show significant improvement. On the contrary, the second, which is an automatic software-based assessment revealed a significant improvement (*p* = 0.0331) which is consistent with a significant reduction in the quantity of pulmonary fibrosis. At 12 months of combined therapy with RTX initially and MMF afterward, a significant increase in FVC percentages were observed (*p* = 0.0093) and of FEV1 values (*p* = 0.0061) as well, compared to baseline. DLCO values remained stable (*p* = 0.3375). Despite the small number of patients and the absence of a control group, the authors felt optimistic about the sequential treatment of RTX followed by MMF in SSc-ILD. The uncontrolled studies of RTX administration in SSc-ILD are shown in Table 3.

**Table 3:** Uncontrolled trials of RTX in SSc-ILD.

**References**	**No of patients**	**Disease**	**RTX scheme**	**Follow up**	**Outcomes**	**Safety of RTX**
Lafyatis et al. (25)	15	SSc	RA scheme	6 m	Stable PFTs and HRCT score	No serious AE
Daoussis et al. (26)	8	SSc	Lymphoma scheme	2 y	Improved PFTs and HRCT score	3 serious infections, 2 infusion reactions
Smith et al. (27)	8	SSc	RA scheme	2 y	Increased FVC, stable DLCO	5 serious AE/1 death
Bosello et al. (28)	20	SSc	RA scheme	2 y	Stable PFTs and HRCT score	4 serious AE
Fraticelli et al. (29)	15	SSc	RA scheme	6 m	Improved FVC, stable DLCO, Imroved HRCT	No serious AE
Vilela et al. (30)	10	SSc	RA scheme	6 m	Stable PFTs	–
Sari et al. (31)	15	SSc	Ra scheme	Variable	Stable PFTs	No serious AE

#### Retrospective Trials in Patients With SSc-ILD

A placebo-controlled trial by Thiebaut et al. studied retrospectively 13 patients with SSc-ILD who were treated with RTX vs. 26 patients with SSc-ILD that were not for a follow up period of 2 years (24). Both FVC and DLCO values did not change significantly after 1 year. FVC showed 72% at baseline and 85% at month 12 (*p* = 0.6) and DLCO values [40% at baseline vs. 49% at month 12 (*p* = 0.9)]. However, after 2 years of follow-up, 7/13 patients treated with RTX improved their FVC by a gain of 12 points, whereas 14 patients not treated with RT? worsened their FVC by −1.5. DLCO values also improved in the RTX-treated group with a gain of 4, while it worsened with a loss of −4.5 in the non-RTX-treated group. A direct comparison between the 2 groups (RTX group vs. non-RTX) revealed a significant improvement of PFTs in RTX-treated patients regarding both FVC percentages (*p* = 0.003) and DLCO percentages (*p* = 0.03). The authors also further analyzed a total of 42 patients (35 from the literature and 7 from their own series). They reported an increase of FVC from 71% at baseline to 84% at 12 months (*p* = 0.0006) and in DLCO from 58% at baseline to 64% at 12 months (*p* = 0.02) in the RTX group.

Vilela et al. reported 10 patients with diffuse SSc treated with RTX (“RA scheme”) that were evaluated after 6 months (30). No significant changes were seen in PFTs in all patients regardless of the patients had early (<4 yrs) or late progressive disease. Sari et al. claimed a beneficial effect of RTX therapy in 14 patients with SSc and chronic extensive restrictive lung disease resistant to previous therapy for 15 months of follow up (31). However, heterogeneity in treatment cycles and in time-points of evaluation limit the value of any potential conclusions of this report.

## A Meta-Analysis of Patients With SSC-ILD

A recent systematic review and meta-analysis by Goswami et al. evaluated the efficacy of RTX treatment on pulmonary function parameters of patients with SSc-ILD (32). Twenty studies were included in this meta-analysis among which there were two randomized controlled trials, six prospective studies, five retrospective studies, and seven conference abstracts. RTX was reportedly associated with increases of FVC by 4.49% (95% CI 0.25, 8.73) and similarly with increases of DLCO by 3.47% (95% CI 0.99, 5.96) at 6 months. Furthermore, FVC values were increased by 7.03% (95% CI 4.37, 9.7) and percentages of DLCO by 4.08% (95% CI 1.51, 6.65) after 1 year of treatment with RTX. Two studies comparing RTX treatment to other immunosuppressive treatments showed improvements in FVC in the RTX group that was 1.03% (95% CI 0.11, 1.94) greater than the improvement of FVC values of patients in the immunosuppressive group at 6 months but not at 12 months. Changes in DLCO values were similar in both groups at both time points. Patients on RTX had lower rates of infection as well. The authors acknowledge another limitation of this meta-analysis such as the different disease traits, RTX administration schemes, and evaluation of PFTs at the standard time points of 6 and 12 months.

## Retrospective Trials in Patients With RA-ILD

Fui et al. studied 28 patients with RA 21 of which had ILD (33). Fourteen of 28 were treated with RTX due to worsening ILD. PFTs and HRCTs were evaluated before and 6 and 12 months later. FVC before RTX administration was 87.11 ± 6.13% and FVC after 12 months of RTX treatment was 93.01 ± 23.01%, *p* = NS. DLCO percentages before RTX treatment were 53.76 ± 5.37% and DLCO 12 months after RTX administration was 54.9 ± 15.2%, *p* = NS. Therefore, we conclude that patients receiving RTX had stabilization of their PFTs. Patients were also separately analyzed as UIP HRCT patterns and NSIP HRCT patterns. For the UIP-ILD pattern, there was a loss of 301 ml of FVC at 1 year, while for the NSIP-ILD the loss was limited to 51 ml compared to baseline volumes, non-significant reductions. However, a direct comparison of volume losses between the UIP and NSIP–ILD volume losses disclosed that reductions seen in the UIP-ILD subgroup were significant. The study included also 14/28 patients with RA that did not receive RTX and were evaluated as well at baseline and 12 months. However, a direct comparison between the RTX and the non-RTX group revealed no significant changes. The patients with RA–UIP pattern had a bad prognosis, despite the observation that PFTs of these patients did not worsen after RTX administration. It is of interest that in this retrospective study another subpopulation not receiving RTX was evaluated and was used as a control subpopulation.

Md Yusof et al. evaluated retrospectively 700 patients with RA with 56/700 diagnosed with ILD (34). Some patients were treated with RTX due to worsening ILD (*n* = 10) or due to unresponsiveness to previous treatment for RA (*n* = 6). PFTs were evaluated in all 56 patients with RA-ILD. A numerical improvement was observed in FVC after 6–12 months of RTX administration compared to baseline from −2.4% pre—RTX treatment to +1.2% post-RTX treatment, with a median difference of +4.2%, *p* = 0.025. DLCO values had an increase from−4.4% pre-RTX to −1.3% post-RTX with a median difference of +3.7%, *p* = 0.045. Fourteen patients with worsening ILD were evaluated with HRCT before and after treatment with RTX. Improvement of HRCT images was seen in one patient (7%), stabilization in six patients (42%), and worsening in seven patients (50%) after RTX administration. A direct comparison between the patients with responsive NSIP-ILD (*n* = 33) and the patients with progressive non-responsive NSIP-ILD revealed that DLCO values declined more in the non-responsive group (−3.8% responsive NSIP-ILD vs. −17.5% non-responsive NSIP-ILD, *p* = 0.037).

In their retrospective study Narvaez et al. analyzed 31 patients with RA that developed serious ILD refractory to the previous DMARD therapy (35). Some patients switched to RTX from other biologic agents (n=12) or had already failed to respond to previous ILD treatment (n=3). HRCTs were performed after RTX treatment, only in those patients that had worsening dyspnea or deterioration of PFTs. RTX treatment prevented the further reduction of FVC at 1 year compared to baseline (delta: percentage change from the start of therapy): Δ% pFVC +8.06% (95% CI: −10.9–−5.2; *p* < 0.001). Moreover, DLCO did not further decline after 1 year compared to baseline Δ% pDLCO: +12.7% (95% CI: −16.3 to −9.1; *p* < 0.001. When the patients were divided into a UIP (*n* = 13) or a non-UIP- pattern (*n* = 18) and analyzed separately, PFTs in both groups improved at 1 year compared to baseline levels. A quantitative ILD score (QILD) score was used in HRCTs to define the extent of lung disease. Eighteen/31 patients had a QILD of 18.6 ± 12% before treatment with RTX. Six of the 18 patients (33%) worsened their QILD score (mean −5% ± 0.7), 2/18 (11%) improved their score (mean +6.5% ± 1.5), and 10/18 (56%) had the same QILD score after 21 months of RTX administration in comparison to baseline QILD score. In addition, 25 patients with RA evaluated at 2 years of treatment with RTX displayed a significant improvement of both FVC and DLCO compared to pretreatment levels.

### Prospective Trials in Patients With RA-ILD

In their study, Matteson et al. evaluated 10 patients with RA-ILD treated with RTX (7/10) for a maximum of 48 weeks (36). By week 48, FVC values declined by at least 10% in 1/7 patients, remained stable in 4/7, and increased by at least 10% in 2/7 patients. DLCO worsened by at least 15% in 1/7 patients, was stable in 4/7 patients, and increased by >15% of baseline in 2/7 patients. HRCT scores improved in 1 patient, worsened in another patient and stabilized in the rest of 5/7 patients assessed at week 48 after RTX administration.

Vadillo et al. enrolled 68 patients from two different medical centers diagnosed with RA ILD (37). Functional impairment was defined as a reduction of FVC < 5%. By the time ILD was diagnosed treatment of patients changed with preferential use of RTX, abatacept, and azathioprine and reduced use of MTX, LEF, and anti-TNFα mAb. Thirty-one out of 68 patients with RA were treated with RTX with a mean follow-up of 20.6 months and maximum exposure of 8.8 years. Multivariate analysis revealed that PFTs were preserved in patients with RA treated with RTX compared to patients on other treatment regimes. The same analysis disclosed that RTX had less risk of deterioration (Hazard Ratio and 95% confidence interval) [HR 0.51 (95% CI 0.31, 0.85), *p* = 0.01] vs. patients with RA on other treatments. The doses of RTX administered, the repetition of cycles as well as the number of cycles of RTX differed in each patient. Despite such confounding factors, the results of this trial could be considered as promising.

## Retrospective Studies in Patients With AS

Marie et al. studied seven patients with AS that manifested with severe ILD that had previously failed treatments with immunosuppressive agents (38). After having received RTX treatment for 1 year, the patients exhibited improvement of PFTs.

FVC percentages increased from a pre-treatment mean of 66% to a post-treatment of 74 %, *p* = 0.04 and an increased DLCO from 39% (pre-treatment) to 59% (post treatment), *p* = 0.001. HRCT scoring disclosed an improvement of ILD abnormalities at 1 year after RTX treatment in 5/7 patients and stabilization of ILD abnormalities in 2/7 patients. The dosages of steroids were tapered by almost 50% of the pretreatment dosages after treatment with RTX. This small uncontrolled and retrospective study favors RTX use in refractory cases of AS—associated ILD.

Twenty-five patients with AS autoAb and ILD treated with RTX were retrospectively evaluated in the study of Doyle et al. (39). RTX was administered because of recurrent or progressive ILD. Twenty-one out of 25 patients were treated with RTX as a second-line medication due to unresponsiveness to previous DMARDs and 4 out of 25 were treated with RTX as a treatment of the first choice. Prednisone and other DMARDs were allowed. PFTs and HRCT scores were evaluated before and 1 year after treatment with RTX. FVC (*n* = 19) was stable or improved by 79% at 1 year after treatment with RTX compared to baseline. In addition, HRCT average scores (*n* = 8) were stable or improved in 88% of subjects. FVC was also statistically increased for seven patients with AS 3 years after RTX treatment compared to pretreatment levels (+21%, *p* = 0.016). DLCO percentages on the contrary were reduced 1 year after treatment with RTX compared to baseline, from 42 ± 17 to 36 ± 16. Nevertheless, 2 years after RTX administration, DLCO percentages increased compared to baseline from 36 ± 16 to 53 ± 26. The authors report that the dosages of glucocorticoids were stable or reduced in 88% of patients. Moreover, it is of importance that the patients who received more than one cycle of RTX (*n* = 17) compared to the patients that received only one cycle of RTX (*n* = 8) had an increase of FVC of 9.3% (*p* = 0.0077). Patients having NSIP patterns in HRCT had a better response to RTX treatment. In addition, patients with mild disease (based on good FVC and DLCO parameters) responded better to treatment with RTX.

Andersson et al. studied retrospectively 24 patients with AS with severe ILD involvement and were administrated RTX (40). During the follow-up period (median: 52 months after RTX treatment), FVC values were increased from 58% at baseline to 72% at post-treatment (*p* < 0.018). DLCO values similarly increased from 41% at baseline to 48% post-treatment, (*p* < 0.025). The extend of ILD-affected lung parenchyma on HRCT was reduced by a median of 33% compared to pre-treatment HRCT scores. The follow-up period after RTX treatment is more than 4 years, which is long enough. Patients treated with RTX that were followed up for <12 months had a significant improvement in PFTs and HRCT extent of ILD after RTX treatment.

Langlois et al. compared CYC (*n* = 32) followed by standard immunosuppressive agents vs. RTX (*n* = 28) treatment administered every 6 months in patients with ASS-related ILD (41). CYC was administered (750 mg/m^2^/month) for 6 months and then was switched to other immunosuppressive drugs (AZA, MMF, MTX). PFTs showed a significantly increased median FVC from 53% at baseline to 62% at 6 months after CYC treatment, *p* = 0.01. DLCO percentages also increased from 31.5% at baseline to 35% post treatment, *p* = 0.01. Additionally, eight patients had HRCT scores improved, 14 stabilized, and eight worsened their HRCT scores at 18 months of follow-up after treatment with CYC. RTX was given in the “RA scheme” every 6 months. FVC increased significantly at 6 months after therapy with RTX compared to baseline from 64 to 74%, *p* = 0.002 and DLCO increased slightly, from 45% at baseline to 48% at 6 months after RTX, *p* = 0.1. FVC values were statistically and significantly higher at 1 and 2 years after RTX treatment compared to pre-RTX values. DLCO percentages did not display significant increases at 1 and 2 years of treatment with RTX compared to baseline. Eleven patients improved their HRCT scores, 12 stabilized and 1 worsened their HRCT scores after RTX administration during a mean follow-up of 17.5 months. Upon comparison of the 2 groups treated with CYC vs. treated with RTX, it was observed that the group that initiated CYC had already lower PFT values compared to the group that initiated RTX. More specifically, FVC was 53% in the CYC group vs. 64% in the RTX group, (*p* = 0.04) and DLCO was 32% in the CYC group vs. 45% in the RTX group (*p* = 0.01), respectively. In addition, it was recorded that RTX was administered in patients with the more refractory disease compared to the cases in that CYC was given. The number of previous immunosuppressive treatments contributed to this conclusion: 2.32 ± 1.45 in the RTX group vs. 1.35 ± 1.39 in the CYC group, (*p* = 0.004), respectively.

RTX and CYC demonstrated similar pulmonary progression-free survival (PFS) at 6 months after treatment (92% RTX vs. 85% CYC, respectively), but RTX was superior to CYC at 2 years of treatment (HR 0.263, 95% CI 0.094–0.732, *p* = 0.011). RTX treatment was superior in the maintenance phase compared with immunosuppressants continued after CYC. No significant differences were reported in the dosage of the steroid and in the adverse events of the two treatments as well. Despite the retrospective nature of this study and the dissimilarity of the patients comprising the 2 treatment groups this study is acknowledged as the first comparison study of RTX vs. other immunosuppressive treatment in AS-ILD.

The majority of the studies of RTX conducted in groups of patients with different CTDs–ILD or in patients with one underlying disease–ILD such as SSc, RA, AS, has noted that B cell depletion treatment was safe and well-tolerated. A few gastrointestinal complaints have been reported and to a lesser extent infusion reactions. Our major concern is focused on infections, especially respiratory tract infections (42).

## Studies in Patients With SS

A few case reports and even fewer retrospective studies have evaluated RTX in ILD in patients with SS (43, 44). Chen et al. studied retrospectively 10 patients with primary SS (pSS) with moderate to severe ILD that had previously been treated with DMARDs (45). PFTs at 6 months after RTX treatment revealed an improvement of DLCO from 49.3 ± 12.6% at baseline to 56.9 ± 11.4% at 6 months after RTX, (*p* = 0.011) but a non-significant change of FVC from 74.7 ± 16.2% at baseline to 76.4 ± 16.1 % at 6 months, (*p* = 0.484). Mean HRCT scores decreased but not significantly after rituximab in 7/10 patients (from 8.7 ± 4.1 to 7.6 ± 4.6, *p* = 0.419). A significant improvement in HRCT imaging was reported only in 1 pSS patient at 6 months. In addition, all 10 pSS patients with ILD had a restrictive pattern with low DLCO (<75%) and 2/10 had a mixed type of obstructive and restrictive patterns (decreased FVC, <60%). According to this observation, RTX may be beneficial for patients with pSS during active phases of ILD.

## SLE

ILD, although uncommon in SLE, is highly associated with increased mortality. ILD in SLE presents as NSIP, OP, LIP, follicular bronchitis, and UIP. No clinical trials and head-to-head studies exist assessing the treatment of SLE-related ILD, let alone with RTX treatment. Only case reports and case series have been reported regarding the effects of RTX treatment on all manifestations of patients with SLE (46).

Acute lupus pneumonitis is also a serious manifestation of SLE as well as pulmonary hemorrhage. Immunosuppressive agents including RTX have been used. RTX has also been employed with promising results in shrinking lung syndrome in patients with SLE as a few case reports have reported (47, 48).

One retrospective study included 11 patients with refractory SLE to the previous use of steroids and immunosuppressive agents (49). The patients were given RTX as an add-on therapy and were evaluated at 6 months after RTX administration. Seven out of 11 patients were simultaneously treated with CYC and 6 out of 11 were treated with IV steroids. Three patients had severe lung disease with significant morbidity. One patient responded well to RTX with improvement in FVC and total lung capacity. Two patients preserved a non-deteriorating respiratory function, and 1 patient had an objective improvement in lung function as he remained stable after RTX treatment. FVC did not change significantly in the whole group of 11 patients, *p* = 0.11. One might assume that RTX halted the deterioration and progression of lung disease in these few patients, whereas previous immunosuppressants had previously failed in halting ILD progression. Table 4 collects all studies mentioned above regarding RTX in RA-, AS-, SS-, and SLE-related-ILD.

**Table 4:** Trials of RTX use in RA/AS/SS/SLE-ILD.

**References**	**No of patients**	**Disease**	**RTX scheme**	**Follow up**	**Outcomes**	**Safety of RTX**
Fui et al. (33)	14 RTX vs. 14 non-RTX	RA	RA scheme	12 m	Stable PFTs and HRCT score vs. reduced PFTs in control	No serious AE
Md Yusof et al. (34)	56	RA	RA scheme	12 m	Increased PFTs/HRCT score	33/56 serious AEs/infections 12/56 deaths
Narvaez et al. (35)	31	RA	RA scheme	12 m	Improved PFTs improved/stable HRCT	Few serious AE 10/31 2/31 deaths
Mattesson et al. (36)	10	RA	RA scheme	12 m	Stable PFTs/stable HRCT	3/7 AEs 2/7 death
Vadillo et al. (37)	31 RTX/37 non-RTX	RA	RA scheme	6 m	Stable PFTs vs. non-stable PFTs in control group	A few AEs
Marie et al. (38)	7	AS	RA scheme	12 m	Improved PFTs/HRCT score/steroid reduction	No serious AE
Doyle et al. (39)	21	AS	RA scheme	12 m	Improved PFTs and HRCT/steroid reduction	No serious AE
Andersson et al. (40)	24	AS	RA scheme	52 m	Improved PFTs and HRCT	6/34 serious infection, 7/34 death
Langlois et al. (41)	28 RTX vs. 32 CYC	AS	RA scheme	2 y	Improved PFTs and HRCT score in both groups	Similar AE
Allenbach et al. (43)	10	AS	RA scheme	1 y	Improve PFTs/stable HRCTscore	No serious AE
Chen et al. (45)	10	SS	RA scheme	6 m	Improved FVC, stable DLCO,Stable HRCTscore	No serious AE
Reynolds et al. (49)	11	SLE	RA scheme (*n* = 9) Lymphoma scheme (*n* = 2)	6 m	Stable PFTs	No serious AE

## Studies in Patients With AAV-ILD

ILD has been related to ANCA-associated vasculitis (AAV) over the past three decades. ILD can be present in 23% of patients with granulomatosis with polyangiitis (GPA) and up to 45% of patients with microscopic polyangiitis (MPA). ILD may precede or manifest concurrently with other manifestations of AAV (50). Radiologic patterns in HRCT include UIP (up to 78% of cases), NSIP (ranging from 13 to 64% of cases), desquamative interstitial pneumonia-like pattern, OP, and combined pulmonary fibrosis and emphysema (CPFE). Although there are treatment guidelines for the initial therapy of patients with AAV, limited data exist for the treatment of patients with AAV (51). RTX is a licensed and commonly used immunosuppressive treatment in patients with AAV-ILD both for induction of, and for maintenance of disease remission.

The study of Maillet et al. evaluated retrospectively 62 patients with AAV-ILD vs. 124 patients with AAV without ILD who were gathered from five prospective trials and were considered as a control group (52). Patients with AAV–ILD received more frequent immunosuppressive treatment for induction and maintenance of disease remission than the control group did. The comparison concerns the two groups of AAV patients, those with ILD and those without ILD. RTX was administrated for induction of remission in 6/62 patients with AAV-ILD and as maintenance therapy in 24/62 patients with AAV-ILD. RTX (27%) and CYC (41%) were most commonly prescribed for re-inducing remission. The prognosis of relapsing disease was good since 95% of patients achieved re-remission. Therefore, this study cannot account for RTX effects in patients with AAV-ILD. RTX was administered as a maintenance treatment more frequently in AAV-ILD than in AAV-non-ILD (40 vs. 4%). Clearly, a large study evaluating RTX-treatment effects strictly in lung involvement in AAV is lacking, despite the proven efficacy of RTX to control AAV systemic manifestations.

## Discussion

The progressive and life-threatening nature of CTD-ILD represents a major challenge. Thus, it is important to identify and apply an aggressive and effective treatment yet with an acceptable safety profile, that will at least slow down disease progression. MMF has been acknowledged as a first-line treatment agent in SSc-ILD. RTX has been employed in a good number of case series and studies both controlled and uncontrolled regarding CTD-ILD in two different treatment approaches. The results of these studies are encouraging given the heterogeneity of patients studied and additional potentially confounding factors that affect the evaluation of PFTs and HRCT scoring methods after RTX administration.

Based on data discussed herein, patients with CTD-ILD, either early- or long-standing disease, mild or severe disease, and resistant disease to previous therapy may represent successful candidates for B cell depletion treatment. In addition, despite a semi-quantitative approach, imaging studies may also depict improvements following RTX treatment. B cell depletion administered in repeated cycles may be a better approach in contrast to a single infusion. It is not entirely clear if it is preferred to employ RTX as a monotherapy or as a combination treatment along with other immunosuppressive/anti-fibrotic medications.

Nevertheless, all existing data point to an additional benefit of RTX treatment, i.e., the steroid-sparing effect. Finally, the use of RTX as a first-line agent and not as an agent used in unresponsive cases represents another unanswered question and an additional challenge in the successful treatment of patients with CTD-ILD.

Published trials suffer from a marked multifactorial heterogeneity: differences in the groups of patients included regarding the CTD disease type, the disease duration, previous and current treatments, the extent, the pattern and chronicity of lung disease, the lack of clearly defined cut-off values for PFT parameters such as the FVC and DLCO, as well as a non-digital scoring system for HRCT lesions, all limit our clear understanding of the benefits expected from B cell depletion treatment in patients with CTD -ILD. Well-designed, large enough studies are needed to overcome such limitations. Despite all the above limitations and downsides, one should keep in mind that the vast majority of studies published so far support a significant improvement of CTD-ILD in patients treated with a B cell depleting therapeutic approach.

## Author Contributions

S-NL conceived, wrote, and edited manuscript. CB wrote and edited manuscript. All authors contributed to the article and approved the submitted version.

## Conflict of Interest

The authors declare that the research was conducted in the absence of any commercial or financial relationships that could be construed as a potential conflict of interest.

## Publisher's Note

All claims expressed in this article are solely those of the authors and do not necessarily represent those of their affiliated organizations, or those of the publisher, the editors and the reviewers. Any product that may be evaluated in this article, or claim that may be made by its manufacturer, is not guaranteed or endorsed by the publisher.
